# Gsmodutils: a python based framework for test-driven genome scale metabolic model development

**DOI:** 10.1093/bioinformatics/btz088

**Published:** 2019-02-13

**Authors:** James Gilbert, Nicole Pearcy, Rupert Norman, Thomas Millat, Klaus Winzer, John King, Charlie Hodgman, Nigel Minton, Jamie Twycross

**Affiliations:** 1 Synthetic Biology Research Centre, University of Nottingham, Nottingham, UK; 2 School of Biosciences, University of Nottingham, Sutton Bonington, Loughborough, UK; 3 School of Mathematical Sciences, University of Nottingham, Nottingham, UK; 4 School of Computer Science, University of Nottingham, Nottingham, UK

## Abstract

**Motivation:**

Genome scale metabolic models (GSMMs) are increasingly important for systems biology and metabolic engineering research as they are capable of simulating complex steady-state behaviour. Constraints based models of this form can include thousands of reactions and metabolites, with many crucial pathways that only become activated in specific simulation settings. However, despite their widespread use, power and the availability of tools to aid with the construction and analysis of large scale models, little methodology is suggested for their continued management. For example, when genome annotations are updated or new understanding regarding behaviour is discovered, models often need to be altered to reflect this. This is quickly becoming an issue for industrial systems and synthetic biotechnology applications, which require good quality reusable models integral to the design, build, test and learn cycle.

**Results:**

As part of an ongoing effort to improve genome scale metabolic analysis, we have developed a test-driven development methodology for the continuous integration of validation data from different sources. Contributing to the open source technology based around COBRApy, we have developed the *gsmodutils* modelling framework placing an emphasis on test-driven design of models through defined test cases. Crucially, different conditions are configurable allowing users to examine how different designs or curation impact a wide range of system behaviours, minimizing error between model versions.

**Availability and implementation:**

The software framework described within this paper is open source and freely available from http://github.com/SBRCNottingham/gsmodutils.

**Supplementary information:**

Supplementary data are available at *Bioinformatics* online.

## 1 Introduction

Stoichiometric constraints based modelling for biological systems has been a mainstay of systems biology for several decades (Fell and Small, 1986; Varma and Palsson, 1994). Given its flexibility, low barrier to entry and requirement only on minimal knowledge regarding the stoichiometry of metabolic networks this structural approach has become an extremely popular method for modelling steady-state behaviour of large, biochemical networks (Kauffman *et al.*, 2003). Such large scale reconstructions are often referred to as genome scale metabolic models (GSMMs), as the processes is significantly aided through the advent of relatively inexpensive genome sequencing (Land *et al.*, 2015; O’Brien *et al.*, 2015). Indeed, owing to their ability to model complex aspects of metabolism, GSMMs have been widely adopted as a standard to elucidate and optimize industrial biotechnology processes (Kim *et al.*, 2017).

The reconstruction of GSMMs is a time consuming process of manual curation that follows a complex protocol to ensure model validity (Thiele and Palsson, 2010). Whilst many popular automated methods exist to construct GSMMs from reference genomes (Henry *et al.*, 2010; Poolman, 2006), there is still a significant amount of manual curation. However, treating the creation of models as an isolated ‘one-off’ event ignores the significant amount of curation that is required for applications such as biotechnology.

As a consequence, a significant amount of work has gone into the management of genome scale models. The BiGG models database (King *et al.*, 2016), for example, exists to provide a standardized repository of validated models that can be shared and reused. Perhaps one of the best examples of a well curated model developed in an iterative manner is yeast-GEM, a model for *Saccharomyces cerevisiae* under continuous development (Sánchez *et al.*, 2018). Similarly, the MetaNetX (Moretti *et al.*, 2016) system exists to provide a standardized namespace and toolchain for GSM analysis. However, in many cases too little focus is placed upon the collaborative design aspect of such models with few mechanisms existing to capture the differences between two model versions, *model deltas*.

Better tools for developing automated reconstructions of genome scale models are always under development. For example, a recent development in the domain of genome scale models is the EMBL-GEMS model repository for automated reconstructions of bacterial species created from NCBI annotations by the CarveMe tool (Machado *et al.*, 2018). However, these tools will often add incorrect reactions, such as aerobic reactions in organisms that only survive in anaerobic environments (Norman *et al.*, 2018b). Furthermore, we feel a specific advantage of genome scale models is that they encode domain specific knowledge that allows contradictions in understanding to be uncovered. For example, an entirely automated process is unlikely to find the correct cofactors involved in reactions under specific conditions.

Furthermore, as with many areas of bioinformatic study the number of available computational tools has become vast. This covers a huge variety of software platforms including the COBRA toolbox for MATLAB (Schellenberger *et al.*, 2011), ScrumPy and COBRApy in python (Ebrahim *et al.*, 2013; Poolman, 2006) with additional tools and libraries such as cameo (Cardoso *et al.*, 2018), OptFlux in Java (Rocha *et al.*, 2010) and SurreyFBA (Gevorgyan *et al.*, 2011). Whilst most of these tools are Open Source and follow standards, such as SBML (Finney and Hucka, 2003), it is often challenging to replicate the initial modelling efforts conducted by authors of papers. Consequently, we feel that software tools are urgently needed to address this issue. Similarly, the archetype design, build, test and learn cycle of synthetic biology heavily relies on bioinformatics software and modelling to improve the production of natural products (Carbonell *et al.*, 2016). In order to speed up the use of bioinformatics tools to produce high value platform chemicals, genome scale models are often used to discover methods for process optimization *in silico*.

For example, many tools such as RetroPath (Carbonell *et al.*, 2014a), XTMS (Carbonell *et al.*, 2014b) and GEM-Path (Campodonico *et al.*, 2014) suggest thousands of potential heterologous pathways. Many of these tools significantly increase the value of genome scale models, for example by coupling commodity production to an organism’s growth (Feist *et al.*, 2010). These tools all suggest major changes to wild-type strains must be tracked and compared to allow models to remain relevant. In effect, mechanisms are required to relate to modified test and production strains.

Similarly, many conventional applications of genome scale models in systems biology have often suffered from unnecessary replication of work due to a lack of adherence to standards (Monk *et al.*, 2014). For example, there are now many independently developed models of *Clostridium acetobutylicum* (Dash *et al.*, 2014; Lee *et al.*, 2008; McAnulty *et al.*, 2012; Senger and Papoutsakis, 2008; Yoo *et al.*, 2015), an organism used in the production of solvents for around a century (Moon *et al.*, 2016; Weizmann, 1919). These models all exist to solve similar biological problems some being updates to the initial base models. However, there has been disagreement over fundamental biochemical properties of this anaerobic organism, notably with the focus on redox balancing (Dash *et al.*, 2014). Such models also include updates based on improved genome annotations and the inclusion of fluxomic, transcriptomic and metabolomic characterizations (Yoo *et al.*, 2015).

Unfortunately, many of the results reported in (Dash *et al.*, 2014; Lee *et al.*, 2008; McAnulty *et al.*, 2012; Senger and Papoutsakis, 2008; Yoo *et al.*, 2015) are difficult to compare or reproduce as the result of a number of issues. Often, model authors do not use a standardized set of identifiers for reaction names [such as the MetaNetX namespace (Moretti *et al.*, 2016)], which makes direct comparison of model structure as well as differences between reactions a challenge. Where models are shared, it is often in non-standard spreadsheet formats, rather than SBML models. Indeed, even in the case of valid SBML models being made available at the time of publication few details are given as to how to run such models for conditions discussed in original articles.

In this paper, we present a software framework geared towards *test-driven* genome scale model development, a concept that is taken directly from good software development practices (Martin, 2002). By this we mean the notion that, as a model is curated to represent biological phenomena, much of the validation can be turned into specific test cases that can be repeated between model versions. We provide an example test case for *Clostridium autoethanogenum*, an organism that has had considerable focus in terms of genome scale models and how a working methodology using the software presented here can reduce repetition of work and improve the reproducibility of results. This article aims to summarize the main objectives of the *gsmodutils* software and we refer the reader to the software user guide for a more detailed exploration of features.

## 2 Improving the design phase of industrial biotechnology

**Fig. 1.**
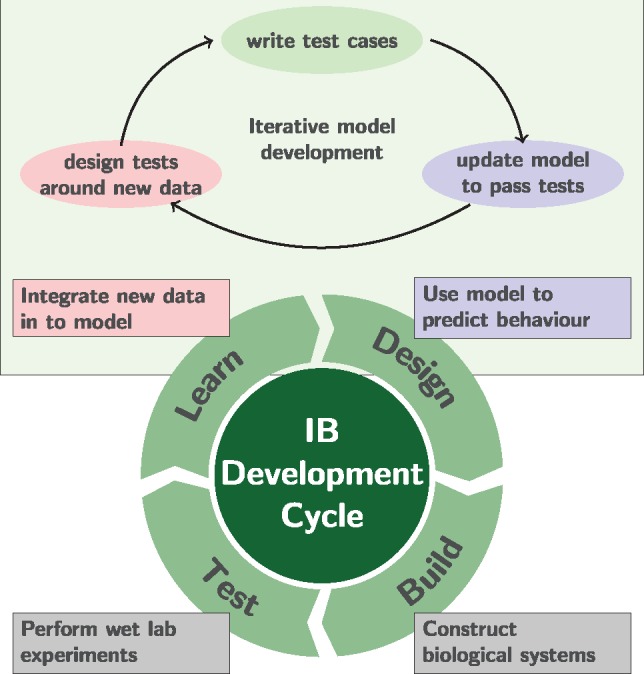
Iterative cycle for systems and synthetic biology development, prevalent in industrial biotechnology applications. This approach captures an iterative mode of development, where models are used to inform wet lab decision making and the information is fed back into future modelling decisions. By integrating test-driven model development (top section) the objective is to simultaneously capture research questions, model validation criteria and minimize the impact of changes on previously completed models

Recent efforts in systems and synthetic biology have been based around a form of iterative, design, build, test and learn cycle (Carbonell *et al.*, 2016) (see Fig. 1). In terms of computational models, this iterative strategy requires adapting and updating models to integrate new biological knowledge (Reed *et al.*, 2006). However, the conventional processes of scientific literature often coalesces to a point at which models are published. In reality, an iterative process means that it is essential that digital experiments can be repeated in a reproducible manner (Cooper *et al.*, 2015). Future changes to models, borne out of a need to meet new challenges and integrate new knowledge, should reflect this.

An *agile* methodology for the development of models places the focus on adapting work to new requirements (Martin, 2002). Such an approach best fits genome scale models because they are rarely created to investigate individual processes and, instead, capture the complexity of large systems. Genome scale models are intrinsically related to available genome annotations. Such annotations rely heavily on automated matches to related species, with the characterizations of individual genes or changes in cofactors and substrates for specific reactions often being left to a few of critical interest (Seemann, 2014). This modelling formation costs an in-depth understanding of dynamic behaviour. However, capturing steady-state phenomena still provides a good understanding of system properties (O’Brien *et al.*, 2015).

As such, approaches often leave models with missing reactions, incorrect gene-reaction rules (Thiele and Palsson, 2010) or with pathways based on gap filling methods that add reactions that may not actually be catalyzed by the organism in question (Benedict *et al.*, 2014). When attempting to understand specific natural phenomena, genome annotations are frequently updated and models are often corrected in an *ad hoc* manner.

Therefore, models undergo significant manual annotation and curation; a process which has a high chance of error. In this work, we advocate a test-driven approach to model development highlighted in Figure 1 (top). Here, the model is changed to achieve research goals that are dynamic in response to the changes of a project. In order to meet this objective, validation criteria for a model such as growth conditions or the impact of gene knock-outs, should be formally set. When a model is changed, all such validation criteria should be retested to ensure that models do not regress to previous states.

We feel that many of the current software tools for genome scale models do an excellent job of facilitating answers to crucial research and design questions. However, there is a major gap in terms of the reliability and re-usability of models due to a lack of standardization and software tools to aid such processes. The following sections provide an overview of the *gsmodutils* software framework. *gsmodutils* aims to provide a basis for test-driven, version controlled agile model development. All software and packages are open source and is designed to be interoperable with platforms widely used in the domain of constraints based modelling.

## 3 Software

### 3.1 Outline and features

Test-driven development is motivated by the idea of clearly defined test cases written before significant changes are made to any underlying architecture. In the case of genome scale models, errors can easily occur as a product of human curation designed to better represent newly discovered aspects of metabolism.

**Fig. 2.**
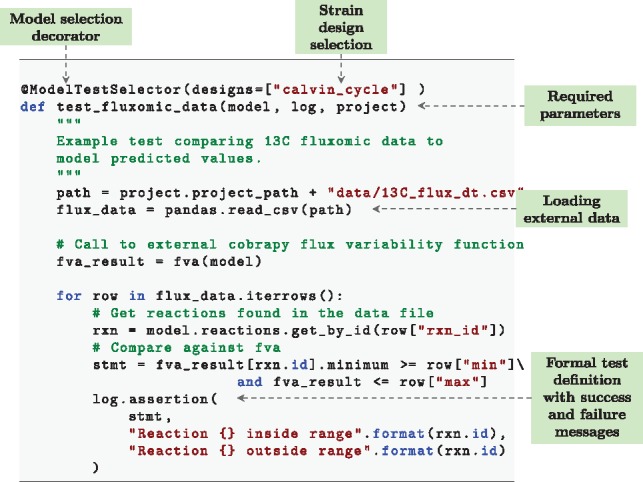
An example *gsmodutils* test case written in python. In this test, flux variability analysis is used to compare a model against ^13^C carbon flux tracking data. The test also demonstrates how designs can be integrated into a test workflow by specifying the identifier in the ‘ModelTestSelector’ function decorator

By automatically integrating COBRApy (Ebrahim *et al.*, 2013) users can easily write convenient test cases following examples given in the user guide. A standard test case, ensuring that a given model grows on media is given in Figure 2. When a new model repository is created with the *gsmodutils* tool, a number of pre-written test cases are automatically added to a file. However, we stress that the vast majority of individual use cases for a model must be specific to a given biological problem.

The software provides a number of features such as import and export of models in different formats and the generation of test reports through use of the command line. The use of flat files enables easy integration with version control software such as git and mercurial. In addition, projects are easy to export using portable standardized docker images (Merkel, 2014), the idea being to allow users to share models as quickly and easily as possible without concern for custom system configurations (see software documentation for more details).

### 3.2 Strain designs

A core aspect behind the implementation of *gsmodutils* is the concept of a *design*, this encompasses a simple set of changes to a ‘wild-type’ model that are required for analysis. However, it is often the case that such deletions are of scientific or industrial interest and, as such, the strain will be used in future work. Consequently, such designs are hereditary in nature. By taking the difference between the constraints applied to an initial model and subsequent modifications, *gsmodutils* allows users to easily reuse and export models with this *design delta*.

Formally, we consider a *design delta* to be the set of differences between any reactions, metabolites and genes stored within the COBRApy object. This should not be considered the same as a *diff* in version control systems such as git. Instead, designs of this nature are stored as JSON objects within the *gsmodutils* project and can be tracked by version control systems.

**Fig. 3.**
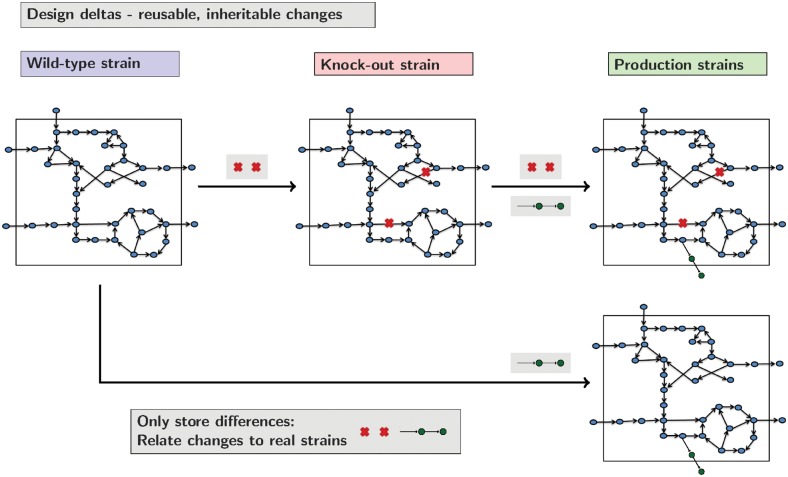
Examples of *gsmodutils* design inheritance. Each design stores the delta between the wild-type base model, any parents and the changes to constraints the design contains. In the example presented above, a heterologous production pathway is combined with a reusable set of knock-outs. Rather than keeping redundant copies of models, designs make projects easier to maintain and understand by only storing annotated differences between models. Designs can then be loaded in a hierarchical manner. In practice, ideally, these designs should relate to experimentally evaluated constructs and strains

As designs inherit from a base model, future curation to a wild-type base model is automatically included in the resulting models. Similarly, designs are self contained and will not interfere with one another allowing project management and annotation as to the function of each design. Figure 3 shows how this could work in a practical situation. Here we consider how functional gene knock-outs can be combined with heterologous genes to create production strains. As designs can be inherited common knock-outs or changes to designs can be combined.

**Fig. 4.**
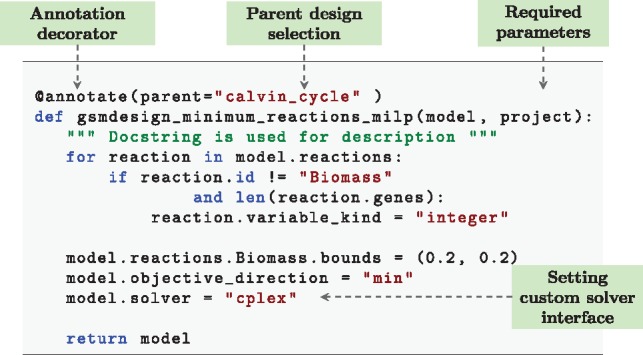
An example *gsmodutils* programmatic design written in python. This design converts reactions to integer type, allowing an MILP formation. The above example seeks to utilize the MILP problem in order to minimize the number of reactions to produce the required biomass components. Loading a model of this form dynamically, as opposed to storing it as an SBML model, allows any underlying reactions to be changed. Designs of this form can also easily be exported to model files via the command line utility

Designs of this nature can also be programmatic, allowing the implementation of features such as non-standard constraints that can be dynamically loaded. An example of this is shown in Figure 4. This example converts an existing model to one based on a Mixed Integer Linear Program (MILP) and sets the objective to minimize the number of enzymatic reactions used with a fixed biomass constraint. This relates to minimizing the number of active genes within a system. As the reaction names do not need to be specified, should reactions be altered within the base model the design will remain functionally the same. Alternative examples could include reductions of models through methods such as elementary flux modes or minimum cut sets, which can change dramatically with only small changes to stoichiometry. Furthermore, functionality of all strain designs is automatically included in tests as part of the default *gsmodutils* testing framework.

### 3.3 Development workflow

In this section, we propose a method for the development of genome scale models that integrates *gsmodutils* with version control systems. The basic workflow is that the user writes a formal test case for some modelling goal, perhaps driven by captured experimental data, that fits a specific form of validation criteria. We note that, in principle, test cases should be written before changes to a model are made.

Figure 2 highlights the notion of test cases, taken from test-driven development. In this example, a reusable test is written that incorperates data from ^13^C metabolic flux tracking. Flux variability analysis is then used to compare the expected flux ranges of a model against the experimental data. The test assertions will pass or fail based upon the models flux values when compared to the experimentally observed data.

## 4 Case study usage Clostridium autoethanogenum

*Clostridium autoethanogenum* is a bacterial species used for the production of commodity chemicals at industrial scale (Abrini *et al.*, 1994; Norman *et al.*, 2018a). A new GSMM of *C.autoethanogenum*, ‘MetaCLAU’, has been analyzed to improve this bioprocess (Norman *et al.*, 2018b) (submitted for peer review). In this section we describe how *gsmodutils* has been utilized to ensure that future versions of MetaCLAU will remain functionally relevant from the perspective of industrial biotechnology.

### 4.1 Scientific background and model integration

*C.autoethanogenum* is a strictly anaerobic, acetogenic bacterium which naturally produces ethanol and trace amounts of 2,3-butanediol (2,3-BD) from carbon monoxide and water (Abrini *et al.*, 1994; Norman *et al.*, 2018a; Schuchmann and Müller, 2014). Since carbon monoxide is readily available in the form of industrial waste gas, and 2,3-BD has a global market value of $43 billion (Köpke *et al.*, 2011), the optimization of yields of 2,3-BD from carbon monoxide is highly desirable in the context of industry (Norman *et al.*, 2018a). MetaCLAU was built using Pathway Tools (Karp *et al.*, 2002) and ScrumPy (Poolman, 2006), and is based on a manually annotated genome sequence of *C.autoethanogenum* (Humphreys *et al.*, 2015). The resulting model consisted of 758 reactions, 773 metabolites and 518 genes. For full details of the model (see Norman *et al.*, 2018b) (submitted for peer review). The model has been integrated with the *gsmodutils* modelling framework as a test-driven project. The following section details specific tests used to evaluate the model at each stage of its continued development.

### 4.2 Evaluation of model validation criteria

In this section we outline specific test criteria that have been applied for the *C.autoethanogenum* model discussed in this study. All of the examples discussed here are available in more detail in the supplementary repository Supplementary Material File S1.

**Energetic consistency:** An important limitation of FBA is that optimal solutions may be thermodynamically infeasible if appropriate constraints are not applied (Fell *et al.*, 2010). In order to identify these unwanted flux distributions and to constrain the model such that they are not in the feasible solution space, a diagnostic FBA is applied with the following constraints: i) All transport reactions are constrained to allow no uptake, and ii) the ATPase reaction is given a fixed flux of one. If a (non-zero) solution to this problem exists, it must contain a thermodynamic inconsistency, which can be dealt with by manual inspection of the solution and modification of one or more of the involved reactions (Fell *et al.*, 2010).

**Flux-minimization tests:** One conventional approach in FBA is to set an optimization criterion of minimizing flux across enzymatic reactions with a fixed biomass constraint (Holzhütter, 2006). The solution to this FBA problem represents minimal protein investment (Holzhütter, 2006). Execution of flux minimization in COBRApy requires a model in which reversible reactions are split into two irreversible reactions, representing forward and backward reactions. A *gsmodutils* strain design was created in which all reversible reactions are split using a programmatic python based design.

In the case of MetaCLAU, the minimal-flux solution includes both ethanol and acetate production, which represents good qualitative agreement with experimental data (Norman *et al.*, 2018a, under review). Since any changes to this predicted phenotype must be investigated, the flux minimization analysis has been formulated as a *gsmodutils* test which utilizes the above mentioned design.

**Product scans:** Of interest to this project were changes in the product spectrum of *C.autoethanogenum* under conditions where the organism can and cannot produce molecular hydrogen (with carbon monoxide as sole carbon and energy source). The hypothesis tested in (Norman *et al.*, 2018a, under review), was that in the case where hydrogen production is infeasible, alternative electron sinks like lactate and 2,3-BD would be produced. As in the previous case, the model-predicted behaviour showing both lactate and 2,3-BD was deemed an important result, which model curators should be notified of if lost during model development. Thus the analysis was built into a *gsmodutils* test.

**Lethal knock-out mutants:** The prediction of lethal single-gene KO mutants through FBA of a GSMM is useful in two ways: i) the identification of essential genes is an important first step for metabolic engineering strategies, and ii) with the advent of high-throughput TraDIS gene-essentiality datasets (Langridge *et al.*, 2009), GSMMs can be validated by their ability to predict essential genes. Furthermore, any change in the set of essential genes (particularly an increase in their number) represents important information for metabolic engineering. For these reasons, a test has been built into the MetaCLAU project which enables the computation of the set of essential genes and their comparison with TraDIS datasets.

## 5 Related software

The reproducibility of computational based research has achieved more and more attention within the last decade (Cooper *et al.* 2015; Peng, 2011; Sandve *et al.*, 2013). Consequently, there has been a proliferation of tools to support researchers in this endeavour. In this section we briefly review a number of tools that exist, both for genome scale models and from the wider mathematical and computational biology community.

**PSAMM:** PSAMM (Steffensen *et al.*, 2016) is a tool that has similar goals to *gsmodutils* in aiding the portability of genome scale models. PSAMM uses a custom YAML format which allows the annotation of models in a simpler manner than the conventional SBML standard. This, simultaneously allows model settings to be easily curated in a human friendly manner as well as allowing changes to be tracked in version control software such as git. This format relates, directly, to a *gsmodutils* design object, which captures the difference between cobra objects. Both approaches allow export to standardized SBML, MATLAB and JSON models for usage in other tools. A core difference between the two frameworks, however, is that PSAMM includes much more functionality for working with genome scale models including gap filling and even its own interface to linear programming solvers. In contrast, *gsmodutils* is designed to sit on top of the already existing COBRApy stack, with tools such as cameo (Cardoso *et al.*, 2018) providing additional functionality for more complex analysis. To this end, *gsmodutils* also has a full python API allowing models and designs to be loaded from within external scripts or jupyter notebooks.

**Memote:** Lieven *et al.* (2018) is an excellent tool with similar ambitions to *gsmodutils* for making reusable genome scale models. It features a fully specified set of tests, including custom test cases and has strong version control integration with git. The core functionality of memote is to provide a standardized, community driven set of tests that check model consistency as well as annotations. Along with user defined tests for experimental data, these tests allow continuous integration as models are updated within a git repository. One of memotes strongest aspects is providing human readable reports between model versions, this allows one to easily track the changes between annotations in reconstructions.

A major difference between these projects is that *gsmodutils* has a stronger focus on reducing the redundancy in model storage through the use of *design deltas*, as described above. Similarly, a core goal of *gsmodutils* is to allow easy import and export outside of the framework for compatibility with other modelling suites. It should be noted that, as memote is written in python, utilizes COBRApy and, at the time of writing, is fully compatible with *gsmodutils*.

**Model repositories:** Models are frequently shared, at the time of publication through services such as BiGG (King *et al.*, 2016) and BioModels (Chelliah *et al.*, 2013). Whilst these repositories encourage the reuse of models and the reproducibility of *in silico* predictions, they are not designed to improve collaboration. The software presented here is designed with the notion that genome scale models are never finished, *per se*, but under continuous development. The cornerstone of this is the use of test cases, which formalize modelling validation criteria. Model repositories such as EMBL-GEMs, based on automated reconstructions generated by CarveMe (Machado *et al.*, 2018), could greatly benefit from an ever evolving set of tests that better capture biological understanding. Indeed, adding more features to control the future management of the BioModels repository has achieved recent attention with tools such as JUMMP (JUst a Model Management Platform (JUMMP) is available at https://bitbucket.org/jummp/jummp/, Accessed: 2018-12-13) that aim to add version control to the management of repositories.

Tools such as BiVes and BudHat (Scharm *et al.*, 2016) also exist and apply more generally than *gsmodutils* to capture the changes to models between versions. In a similar manner to *gsmodutils*, this utility lends itself to version control between model versions by capturing changes to parameters that impact a models performance. As with *gsmodutils*, this type of approach ignores irrelevant properties such as changes to the white space within XML files captured by a conventional unix diff.

## 6 Discussion

In order to facilitate the sharing and dissemination of high quality computational research, good standards and software are required (Jimenez *et al.*, 2017). Naturally a great deal of effort has gone into producing high quality systems and synthetic biology standards (Cox *et al.*, 2018; Hucka *et al.*, 2003). Furthermore, when research projects end it is common for important, large models to be published and become relics lost within the literature, forgotten to all but the most dedicated of individuals. As GSMMs grow in terms of the information about metabolism they contain as well as the biological problems they are used to solve, problems with annotation and curation naturally accumulate as a product of human error. Software that facilitates actively improving how researchers develop and apply models to new phenomena is required.

We have presented a framework with a number of features taken from the software development world specifically designed to improve collaboration and minimize such error. However, it is important to stress the difference between defined behaviour expected from pre-written test cases and novel predictions made by a model. Indeed, a core objective of this framework is to ensure that good practices are followed in model development that help scientists to better trust the results discovered by their models. In an ideal world, we would envision a methodology such as ours becoming a pre-requisite for GSMMs to pass peer review.

As with most software development projects, *gsmodutils* will see expanded features. Initially this will include tighter integration with version control systems such as git and mercurial. Furthermore, the objective of the project is to cultivate collaboration by simplifying the process of distributing large models to different users.

## Supplementary Material

btz088_Supplementary_File

## References

[btz088-B1] Abrini J. et al (1994) *Clostridium autoethanogenum*, sp. nov., an anaerobic bacterium that produces ethanol from carbon monoxide. Arch. Microbiol., 161, 345–351.

[btz088-B2] Benedict M.N. et al (2014) Likelihood-based gene annotations for gap filling and quality assessment in genome-scale metabolic models. PLoS Comput. Biol., 10, e1003882. 25329157 10.1371/journal.pcbi.1003882 PMC4199484

[btz088-B3] Campodonico M.A. et al (2014) Generation of an atlas for commodity chemical production in *Escherichia coli* and a novel pathway prediction algorithm, gem-path. Metab. Eng., 25, 140–158. 25080239 10.1016/j.ymben.2014.07.009

[btz088-B4] Carbonell P. et al (2014a) Retropath: automated pipeline for embedded metabolic circuits. ACS Synth. Biol., 3, 565–577. 24131345 10.1021/sb4001273

[btz088-B5] Carbonell P. et al (2014b) Xtms: pathway design in an extended metabolic space. Nucleic Acids Res., 42, W389–W394. 24792156 10.1093/nar/gku362 PMC4086079

[btz088-B6] Carbonell P. et al (2016) Bioinformatics for the synthetic biology of natural products: integrating across the design–build–test cycle. Nat. Prod. Rep., 33, 925. 27185383 10.1039/c6np00018e PMC5063057

[btz088-B7] Cardoso J.G. et al (2018) Cameo: a python library for computer aided metabolic engineering and optimization of cell factories. ACS Synth. Biol., 7, 1163–1166. 29558112 10.1021/acssynbio.7b00423

[btz088-B8] Chelliah V. et al (2013) Biomodels database: a repository of mathematical models of biological processes. Methods Mol. Biol., 1021, 189–199. 23715986 10.1007/978-1-62703-450-0_10

[btz088-B9] Cooper J. et al (2015) A call for virtual experiments: accelerating the scientific process. Progress Biophys. Mol. Biol., 117, 99–106. 10.1016/j.pbiomolbio.2014.10.001 25433232

[btz088-B10] Cox R.S. et al (2018) Synthetic biology open language visual (sbol visual) version 2.0. J. Integr. Bioinf., 15, 20170074. 10.1515/jib-2017-0074 PMC6167035 29549707

[btz088-B11] Dash S. et al (2014) Capturing the response of clostridium acetobutylicum to chemical stressors using a regulated genome-scale metabolic model. Biotechnol. Biofuels, 7, 144. 25379054 10.1186/s13068-014-0144-4 PMC4207355

[btz088-B12] Ebrahim A. et al (2013) Cobrapy: constraints-based reconstruction and analysis for python. BMC Syst. Biol., 7, 74. 23927696 10.1186/1752-0509-7-74 PMC3751080

[btz088-B13] Feist A.M. et al (2010) Model-driven evaluation of the production potential for growth-coupled products of *Escherichia coli*. Metab. Eng., 12, 173–186. 19840862 10.1016/j.ymben.2009.10.003 PMC3125152

[btz088-B14] Fell D.A., Small J.R. (1986) Fat synthesis in adipose tissue. An examination of stoichiometric constraints. Biochem. J., 238, 781–786. 3800960 10.1042/bj2380781 PMC1147204

[btz088-B15] Fell D.A. et al (2010) Building and analysing genome-scale metabolic models. Biochem. Soc. Trans., 38, 1197–1201. 20863283 10.1042/BST0381197

[btz088-B16] Finney A., Hucka M. (2003) Systems biology markup language: level 2 and beyond. Biochem. Soc. Trans., 31, 1472–1473. 14641091 10.1042/bst0311472

[btz088-B17] Gevorgyan A. et al (2011) SurreyFBA: a command line tool and graphics user interface for constraint-based modeling of genome-scale metabolic reaction networks. Bioinformatics, 27, 433–434. 21148545 10.1093/bioinformatics/btq679

[btz088-B18] Henry C.S. et al (2010) High-throughput generation, optimization and analysis of genome-scale metabolic models. Nat. Biotechnol., 28, 977–982. 20802497 10.1038/nbt.1672

[btz088-B19] Holzhütter H.-G. (2006) The generalized flux-minimization method and its application to metabolic networks affected by enzyme deficiencies. Biosystems, 83, 98–107. 16229937 10.1016/j.biosystems.2005.04.008

[btz088-B20] Hucka M. et al (2003) The systems biology markup language (SBML): a medium for representation and exchange of biochemical network models. Bioinformatics, 19, 524–531. 12611808 10.1093/bioinformatics/btg015

[btz088-B21] Humphreys C.M. et al (2015) Whole genome sequence and manual annotation of *Clostridium autoethanogenum*, an industrially relevant bacterium. BMC Genomics, 16, 1. 26692227 10.1186/s12864-015-2287-5 PMC4687164

[btz088-B22] Jimenez R.C. et al (2017) Four simple recommendations to encourage best practices in research software. F1000Research, 6, 876. 10.12688/f1000research.11407.1 PMC5490478 28751965

[btz088-B23] Karp P.D. et al (2002) The pathway tools software. Bioinformatics, 18, S225–S232. 12169551 10.1093/bioinformatics/18.suppl_1.s225

[btz088-B24] Kauffman K.J. et al (2003) Advances in flux balance analysis. Curr. Opin. Biotechnol., 14, 491–496. 14580578 10.1016/j.copbio.2003.08.001

[btz088-B25] Kim W.J. et al (2017) Current state and applications of microbial genome-scale metabolic models. Curr. Opin. Syst. Biol., 2, 10–18.

[btz088-B26] King Z.A. et al (2016) Bigg models: a platform for integrating, standardizing and sharing genome-scale models. Nucleic Acids Res., 44, D515–D522. 26476456 10.1093/nar/gkv1049 PMC4702785

[btz088-B27] Köpke M. et al (2011) 2, 3-butanediol production by acetogenic bacteria, an alternative route to chemical synthesis, using industrial waste gas. Appl. Environ. Microbiol., 77, 5467–5475. 21685168 10.1128/AEM.00355-11 PMC3147483

[btz088-B28] Land M. et al (2015) Insights from 20 years of bacterial genome sequencing. Funct. Integr. Genomics, 15, 141–161. 25722247 10.1007/s10142-015-0433-4 PMC4361730

[btz088-B29] Langridge G.C. et al (2009) Simultaneous assay of every *Salmonella Typhi* gene using one million transposon mutants. Genome Res., 19, 2308–2316. 19826075 10.1101/gr.097097.109 PMC2792183

[btz088-B30] Lee J. et al (2008) Genome-scale reconstruction and in silico analysis of the *Clostridium acetobutylicum* atcc 824 metabolic network. Appl. Microbiol. Biotechnol., 80, 849–862. 18758767 10.1007/s00253-008-1654-4

[btz088-B31] Lieven C. et al (2018) Memote: a community-driven effort towards a standardized genome-scale metabolic model test suite. bioRxiv, doi: 10.1101/350991/350991.

[btz088-B32] Machado D. et al (2018) Fast automated reconstruction of genome-scale metabolic models for microbial species and communities. Nucleic Acids Res., 46, 7542–7553. 30192979 10.1093/nar/gky537 PMC6125623

[btz088-B33] Martin R.C. (2002) Agile Software Development: Principles, Patterns, and Practices. Prentice Hall, Upper Saddle River, New Jersey.

[btz088-B34] McAnulty M.J. et al (2012) Genome-scale modeling using flux ratio constraints to enable metabolic engineering of clostridial metabolism in silico. BMC Syst. Biol., 6, 42. 22583864 10.1186/1752-0509-6-42 PMC3495714

[btz088-B35] Merkel D. (2014) Docker: lightweight linux containers for consistent development and deployment. Linux J., 2014, 2.

[btz088-B36] Monk J. et al (2014) Optimizing genome-scale network reconstructions. Nat. Biotechnol., 32, 447–452. 24811519 10.1038/nbt.2870

[btz088-B37] Moon H.G. et al (2016) One hundred years of clostridial butanol fermentation. FEMS Microbiol. Lett., 363, doi:10.1093/femsle/fnw001. 10.1093/femsle/fnw001 26738754

[btz088-B38] Moretti S. et al (2016) MetaNetX/MNXref–reconciliation of metabolites and biochemical reactions to bring together genome-scale metabolic networks. Nucleic Acids Res., 44, D523–D526. 26527720 10.1093/nar/gkv1117 PMC4702813

[btz088-B39] Norman R.O. et al (2018a) Progress towards platform chemical production using *Clostridium autoethanogenum*. Biochem. Soc. Trans., 46, 523–535. 29666216 10.1042/BST20170259

[btz088-B40] Norman R.O.J. et al (2018b) A Genome-scale Model of Clostridium Autoethanogenum Reveals Optimal Bioprocess Conditions for High-value Chemical Production from Carbon Monoxide. [UNDER REVIEW].

[btz088-B41] O’Brien E.J. et al (2015) Using genome-scale models to predict biological capabilities. Cell, 161, 971–987. 26000478 10.1016/j.cell.2015.05.019 PMC4451052

[btz088-B42] Peng R.D. (2011) Reproducible research in computational science. Science, 334, 1226–1227. 22144613 10.1126/science.1213847 PMC3383002

[btz088-B43] Poolman M. (2006) Scrumpy: metabolic modelling with python. IEEE Proceed. Syst. Biol., 153, 375–378. 10.1049/ip-syb:20060010 16986321

[btz088-B44] Reed J.L. et al (2006) Towards multidimensional genome annotation. Nat. Rev. Genet., 7, 130. 16418748 10.1038/nrg1769

[btz088-B45] Rocha I. et al (2010) OptFlux: an open-source software platform for in silico metabolic engineering. BMC Syst. Biol., 4, 45. 20403172 10.1186/1752-0509-4-45 PMC2864236

[btz088-B46] Sandve G.K. et al (2013) Ten simple rules for reproducible computational research. PLoS Comput. Biol., 9, e1003285. 24204232 10.1371/journal.pcbi.1003285 PMC3812051

[btz088-B47] Scharm M. et al (2016) An algorithm to detect and communicate the differences in computational models describing biological systems. Bioinformatics, 32, 563–570. 26490504 10.1093/bioinformatics/btv484 PMC4743622

[btz088-B48] Schellenberger J. et al (2011) Quantitative prediction of cellular metabolism with constraint-based models: the Cobra Toolbox v2.0. Nat. Protoc., 6, 1290. 21886097 10.1038/nprot.2011.308 PMC3319681

[btz088-B49] Schuchmann K., Müller V. (2014) Autotrophy at the thermodynamic limit of life: a model for energy conservation in acetogenic bacteria. Nat. Rev. Microbiol., 12, 809. 25383604 10.1038/nrmicro3365

[btz088-B50] Seemann T. (2014) Prokka: rapid prokaryotic genome annotation. Bioinformatics, 30, 2068–2069.24642063

[btz088-B51] Senger R.S., Papoutsakis E.T. (2008) Genome-scale model for *Clostridium acetobutylicum*: part i. metabolic network resolution and analysis. Biotechnol. Bioeng., 101, 1036–1052. 18767192 10.1002/bit.22010 PMC2760220

[btz088-B52] Sánchez B.J. and Jens N. (2015) Genome scale models of yeast: towards standardized evaluation and consistent omic integration. Integrative Biology, 7, 846–858. 26079294 10.1039/c5ib00083a

[btz088-B53] Steffensen J.L. et al (2016) PSAMM: a portable system for the analysis of metabolic models. PLoS Comput. Biol., 12, e1004732. 26828591 10.1371/journal.pcbi.1004732 PMC4734835

[btz088-B54] Thiele I., Palsson B.Ø. (2010) A protocol for generating a high-quality genome-scale metabolic reconstruction. Nat. Protoc., 5, 93. 20057383 10.1038/nprot.2009.203 PMC3125167

[btz088-B55] Varma A., Palsson B.O. (1994) Stoichiometric flux balance models quantitatively predict growth and metabolic by-product secretion in wild-type *Escherichia coli* w3110. Appl. Environ. Microbiol., 60, 3724–3731. 7986045 10.1128/aem.60.10.3724-3731.1994 PMC201879

[btz088-B56] Weizmann C. (1919) Production of acetone and alcohol by bacteriological processes. US Patent 1, 315, 585.

[btz088-B57] Yoo M. et al (2015) A quantitative system-scale characterization of the metabolism of *Clostridium acetobutylicum*. MBio, 6, e01808–e01815. 26604256 10.1128/mBio.01808-15 PMC4669385

